# Supplementary material for: Hog1/p38 regulates the integrated stress response (ISR) and global transcriptomic changes in C. neoformans during oxidative stress
Source: bioRxiv. 2025 May 23:2025.05.19.654802. Preprint. [Version 1] doi: 10.1101/2025.05.19.654802 (PMC12139735; doi:10.1101/2025.05.19.654802)
Supplement: Supplement 1 [file NIHPP2025.05.19.654802v1-supplement-1.pdf]

bioRxiv preprint doi: https://doi.org/10.1101/2025.05.19.654802; this version posted May 23, 2025. The copyright holder for this preprint (which was not certified by peer review) is the author/funder, who has granted bioRxiv a license to display the preprint in perpetuity. It is made available under aCC-BY 4.0 International license.

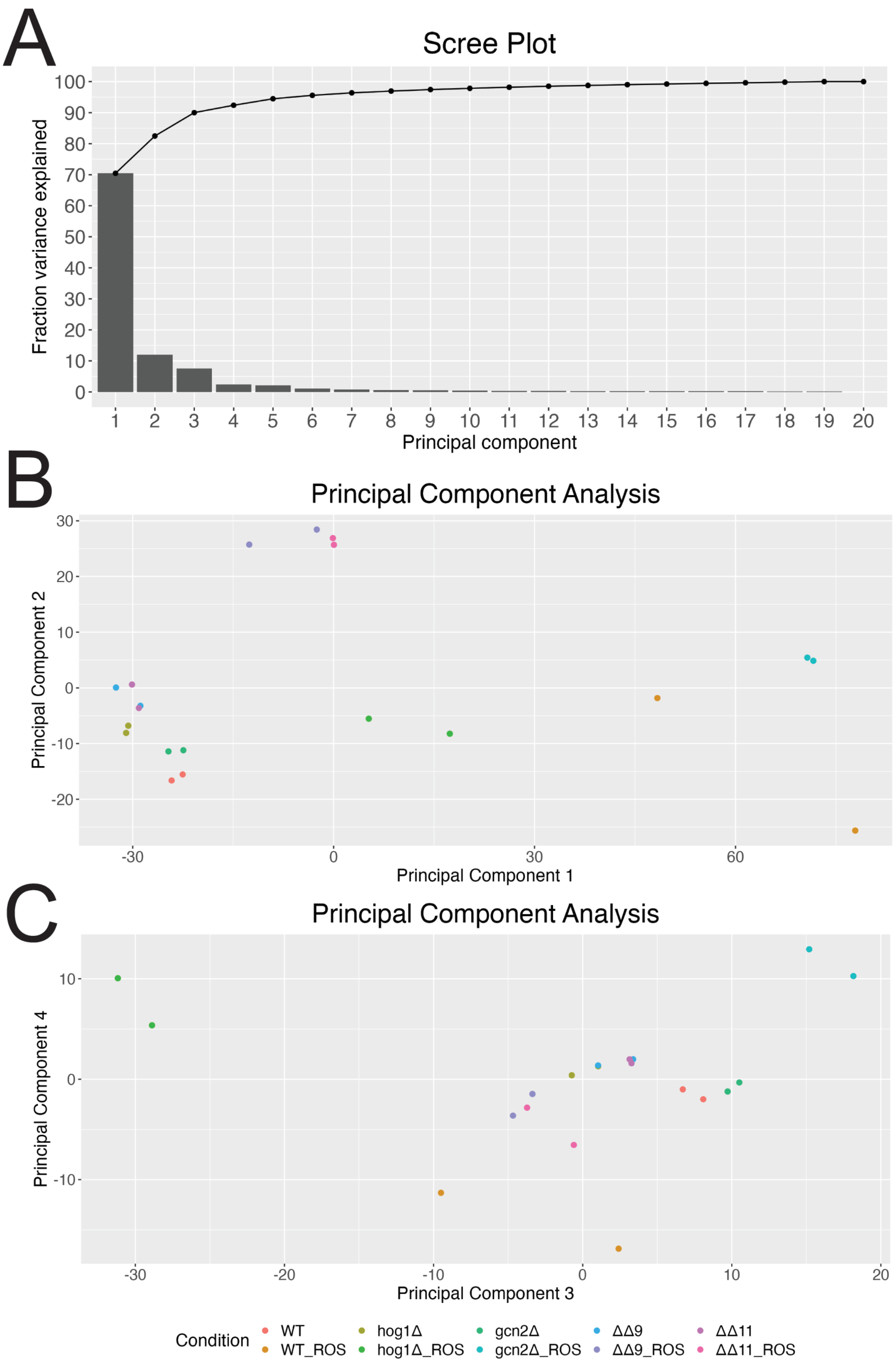

bioRxiv preprint doi: https://doi.org/10.1101/2025.05.19.654802; this version posted May 23, 2025. The copyright holder for this preprint (which was not certified by peer review) is the author/funder, who has granted bioRxiv a license to display the preprint in perpetuity. It is made available under aCC-BY 4.0 International license.

Figure S1: PCA of mRNA-seq data.

PCA was performed on VST-transformed counts for the indicated samples using R. (A) Scree plot indicates the cumulative distribution by which principal components account for variability in the data. (B) PCA plot of Principal Components 1 and 2. (C) PCA plot of Principal Components 3 and 4. Samples with strain name alone are indicative of untreated control samples. Samples with names appended "_ROS" are indicative of cells treated with 2mM H_2O_2 for 1 hour.

bioRxiv preprint doi: https://doi.org/10.1101/2025.05.19.654802; this version posted May 23, 2025. The copyright holder for this preprint (which was not certified by peer review) is the author/funder, who has granted bioRxiv a license to display the preprint in perpetuity. It is made available under aCC-BY 4.0 International license.

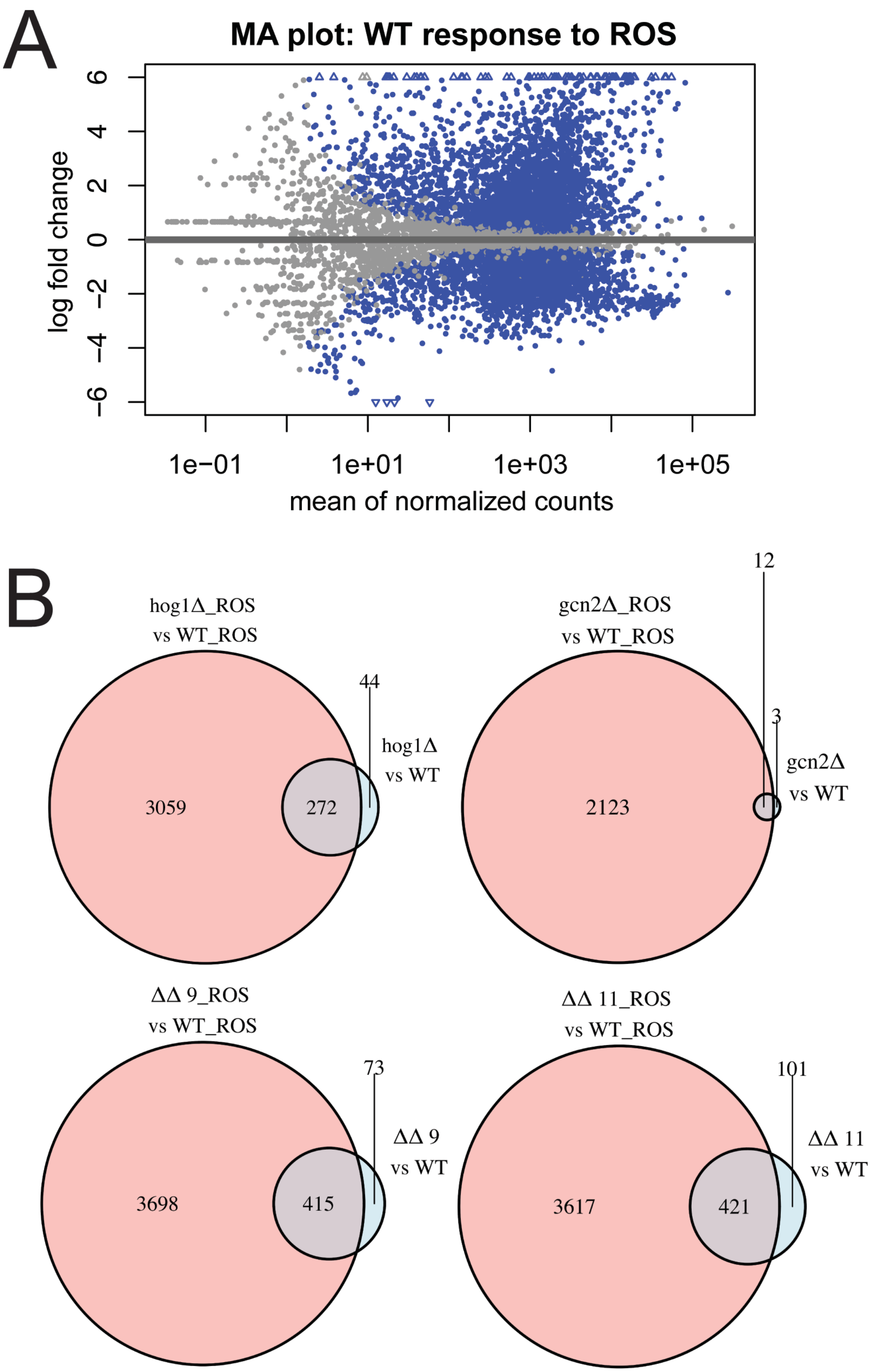

Figure S2: The *C. neoformans* ROS response involves robust changes to gene expression.

bioRxiv preprint doi: https://doi.org/10.1101/2025.05.19.654802; this version posted May 23, 2025. The copyright holder for this preprint (which was not certified by peer review) is the author/funder, who has granted bioRxiv a license to display the preprint in perpetuity. It is made available under aCC-BY 4.0 International license.

(A) MA plot showing the WT response to ROS, with normalized counts on the x-axis and log2FoldChange on the y-axis. Blue dots represent significant changes to gene expression. (B) Summary of differential expression data between mutant strains and WT. Each set of Venn diagrams represents one strain, and compares the number of differentially expressed genes under untreated vs. treated conditions. Samples with names appended "_ROS" are indicative of cells treated with 2mM H_2O_2 for 1 hour.

bioRxiv preprint doi: https://doi.org/10.1101/2025.05.19.654802; this version posted May 23, 2025. The copyright holder for this preprint (which was not certified by peer review) is the author/funder, who has granted bioRxiv a license to display the preprint in perpetuity. It is made available under aCC-BY 4.0 International license.

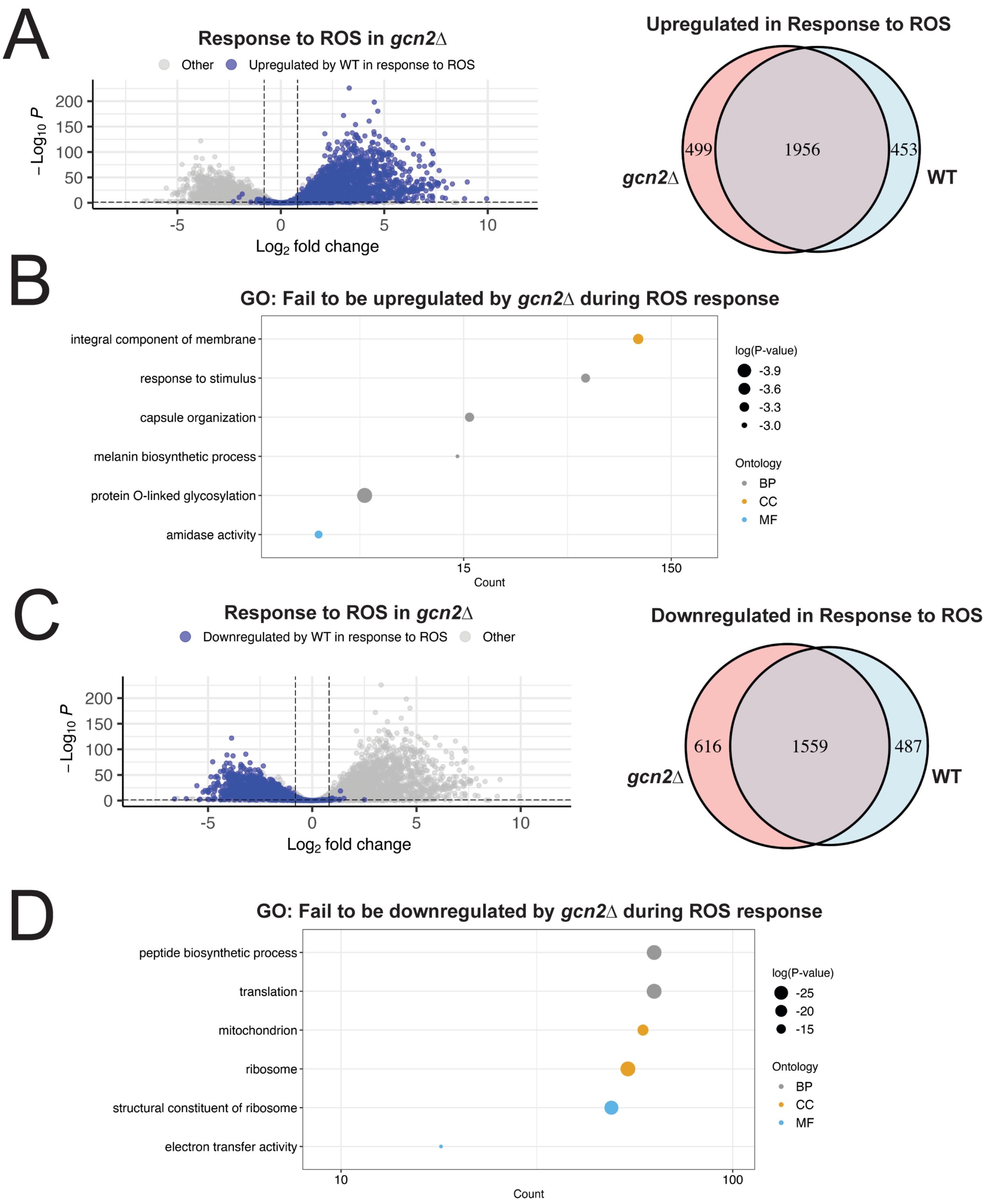

Figure S3: Comparison of the WT and *gcn2Δ* response to ROS.
(A, left) Volcano plot showing the *gcn2Δ* response to treatment with 2mM H_2O_2, with genes upregulated in the WT response highlighted in blue. Dashed lines represent thresholds for differential expression as described in the methods. (A, right) Venn diagram comparing genes upregulated in the WT and *gcn2Δ* responses to ROS. (B) Transcripts that were unique to the WT response (i.e. transcripts that *gcn2Δ* failed to upregulate) were analyzed by GO and visualized as described in Figure 3. (C-D) The same analysis as in *(A-B)*, except focusing on transcripts that were downregulated in the stress response of each strain.

bioRxiv preprint doi: https://doi.org/10.1101/2025.05.19.654802; this version posted May 23, 2025. The copyright holder for this preprint (which was not certified by peer review) is the author/funder, who has granted bioRxiv a license to display the preprint in perpetuity. It is made available under aCC-BY 4.0 International license.

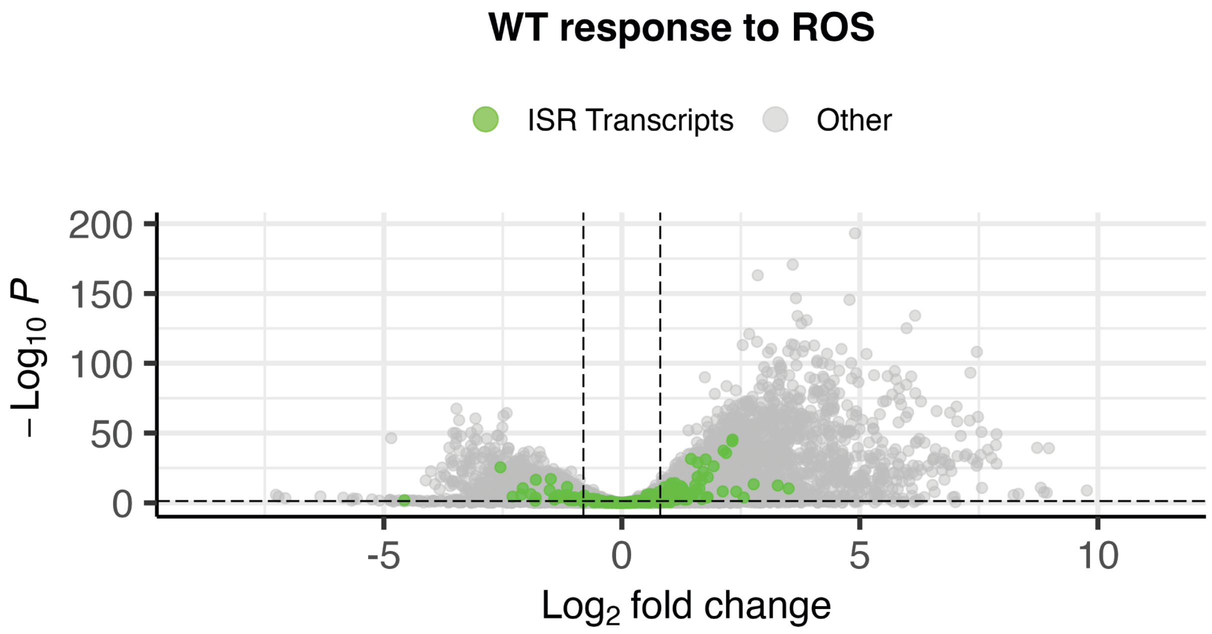

Figure S4: Visualization of the ISR in the WT response to 2mM H_2O_2.
The WT response to treatment with 2mM H_2O_2 was visualized by volcano plot, with transcripts identified as targets of the ISR in a previous analysis highlighted in green. Dashed lines represent thresholds for differential expression as described in the methods.

bioRxiv preprint doi: https://doi.org/10.1101/2025.05.19.654802; this version posted May 23, 2025. The copyright holder for this preprint (which was not certified by peer review) is the author/funder, who has granted bioRxiv a license to display the preprint in perpetuity. It is made available under aCC-BY 4.0 International license.

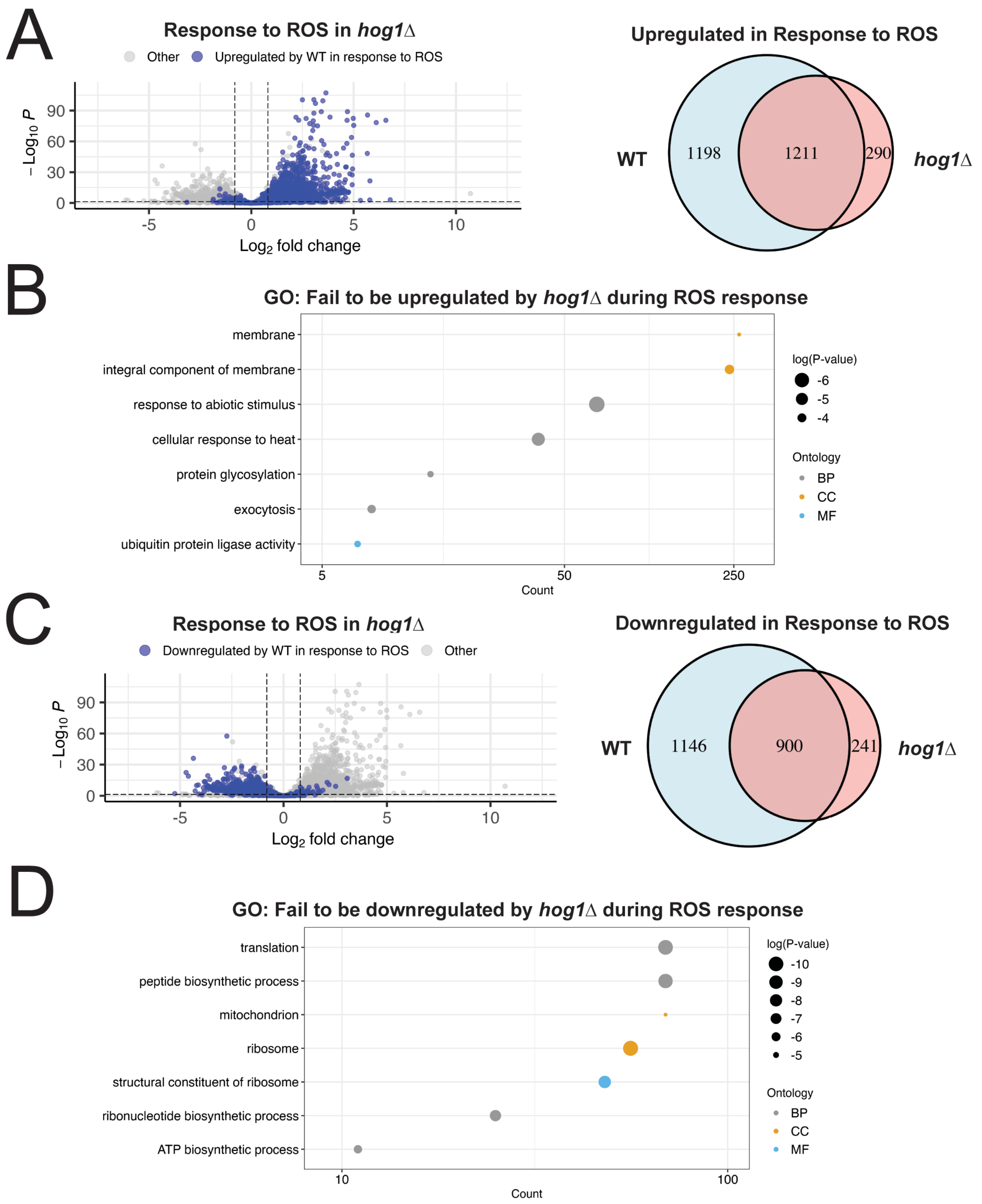

Figure S5: Comparison of the WT and *hog1*Δ response to ROS.
(A, left) Volcano plot showing the *hog1*Δ response to treatment with 2mM H_2O_2, with genes upregulated in the WT response highlighted in blue. Dashed lines represent thresholds for differential expression as described in the methods. (A, right) Venn diagram comparing genes upregulated in the WT and *hog1*Δ

bioRxiv preprint doi: https://doi.org/10.1101/2025.05.19.654802; this version posted May 23, 2025. The copyright holder for this preprint (which was not certified by peer review) is the author/funder, who has granted bioRxiv a license to display the preprint in perpetuity. It is made available under aCC-BY 4.0 International license.

responses to ROS. (B) Transcripts that were unique to the WT response (i.e. transcripts that *hog1*Δ failed to upregulate) were analyzed by GO and visualized as described in Figure 3. (C-D) The same analysis as in A-B, except focusing on transcripts that were downregulated in the stress response of each strain.

bioRxiv preprint doi: https://doi.org/10.1101/2025.05.19.654802; this version posted May 23, 2025. The copyright holder for this preprint (which was not certified by peer review) is the author/funder, who has granted bioRxiv a license to display the preprint in perpetuity. It is made available under aCC-BY 4.0 International license.

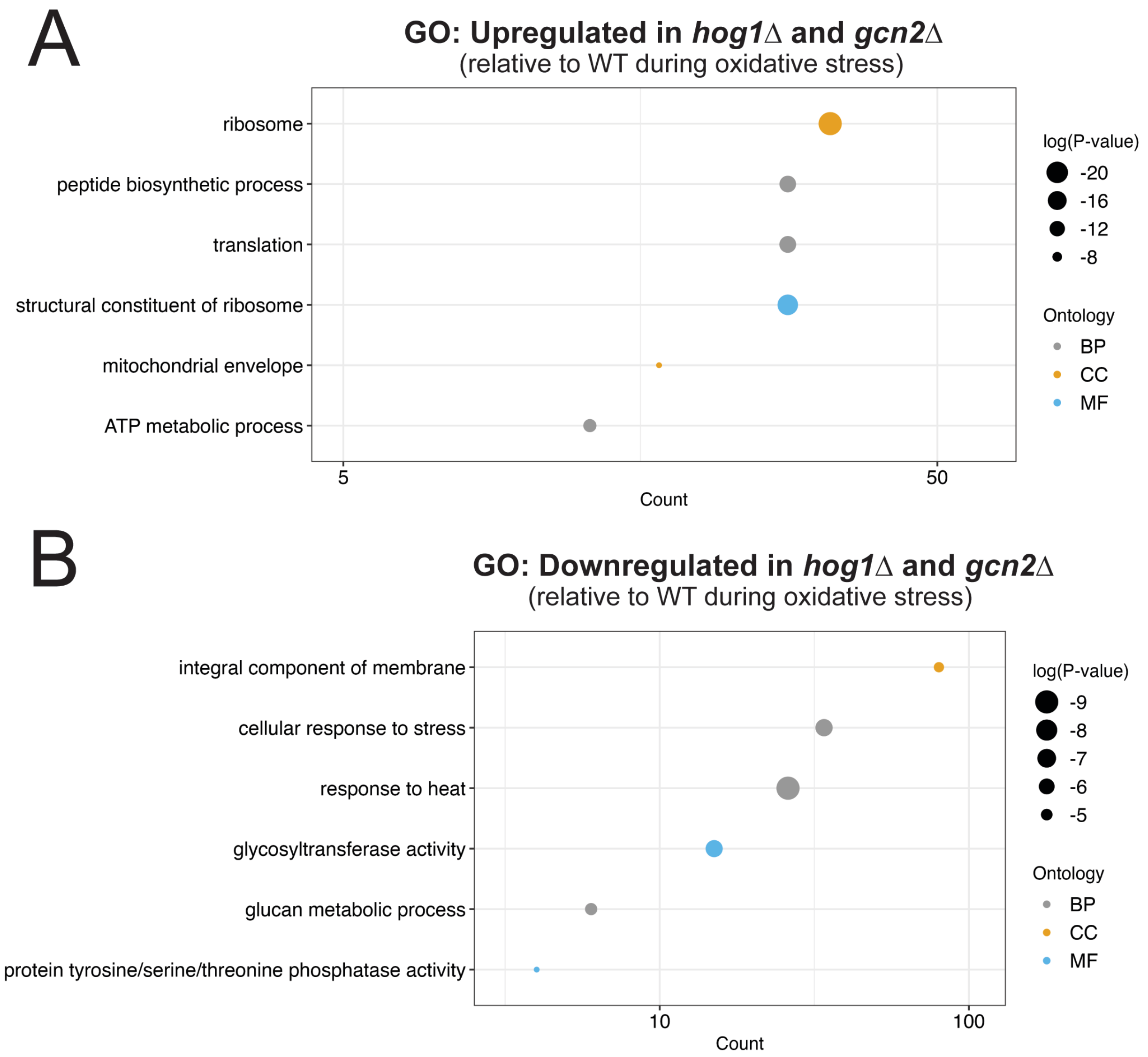

Figure S6: GO analysis of transcripts co-regulated by Hog1 and Gcn2 during ROS.

(A-B) GO analysis was performed on transcripts differentially expressed in both *hog1*Δ and *gcn2*Δ, with respect to WT during ROS treatment. (A) GO analysis of transcripts upregulated in both *hog1*Δ and *gcn2*Δ, with respect to WT during ROS treatment was visualized as described in Figure 3. (B) GO analysis of transcripts downregulated in both *hog1*Δ and *gcn2*Δ, with respect to WT during ROS treatment was visualized as described in Figure 3.

bioRxiv preprint doi: https://doi.org/10.1101/2025.05.19.654802; this version posted May 23, 2025. The copyright holder for this preprint (which was not certified by peer review) is the author/funder, who has granted bioRxiv a license to display the preprint in perpetuity. It is made available under aCC-BY 4.0 International license.

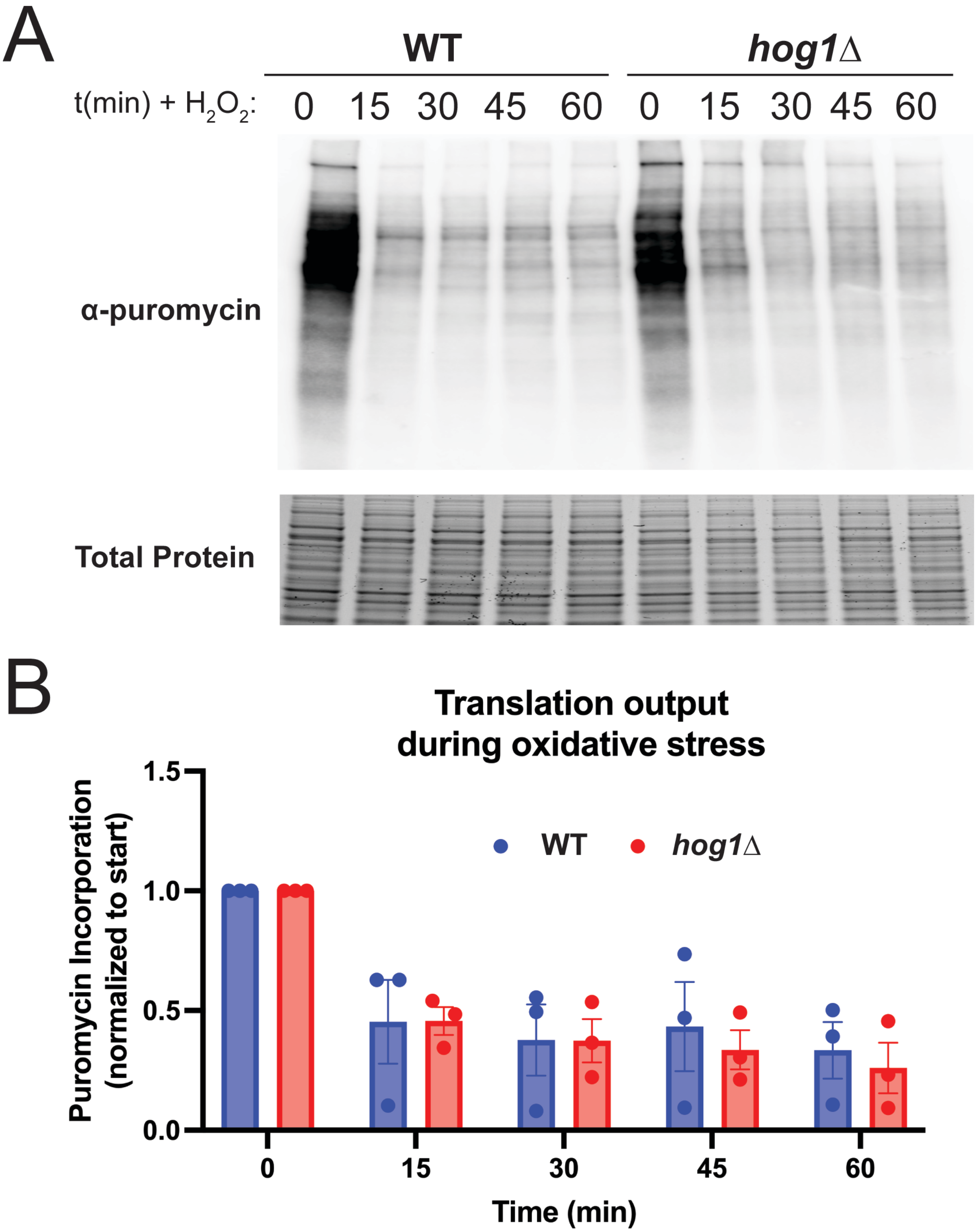

Figure S7: Puromycin incorporation assay in WT and *hog1*Δ during oxidative stress. (A-B) WT and *hog1*Δ were grown to midlogarithmic phase and subjected to 2mM H_2O_2 for the indicated amounts of time. Puromycin was added and incorporated for 10 minutes prior to harvest of each time point. (A) Protein lysates were prepared and probed for puromycin using an anti-puromycin antibody.

bioRxiv preprint doi: https://doi.org/10.1101/2025.05.19.654802; this version posted May 23, 2025. The copyright holder for this preprint (which was not certified by peer review) is the author/funder, who has granted bioRxiv a license to display the preprint in perpetuity. It is made available under aCC-BY 4.0 International license.

Total protein is shown as a loading control. (B) Results from three biological replicates were quantified by densitometry, using total protein signal as the denominator. Then signal for each strain was normalized to the untreated ($t = 0$) sample.

bioRxiv preprint doi: https://doi.org/10.1101/2025.05.19.654802; this version posted May 23, 2025. The copyright holder for this preprint (which was not certified by peer review) is the author/funder, who has granted bioRxiv a license to display the preprint in perpetuity. It is made available under aCC-BY 4.0 International license.

Gene ID	Log_2(fold-change) in Expression		Gene Description
	WT (ROS-treated vs. untreated)	*hog1*Δ vs. WT (during ROS)	
**CNAG_00575**	3.98	-4.38	Catalase 3
**CNAG_00654**	6.61	-3.19	sulfiredoxin
**CNAG_01019**	-0.33	0.15	superoxide dismutase [Cu-Zn]
**CNAG_01138**	3.40	-3.05	cytochrome c peroxidase, mitochondrial
**CNAG_02147**	-1.20	1.07	cytochrome c peroxidase
**CNAG_02399**	0.86	-1.21	glutathione-disulfide reductase
**CNAG_02503**	1.00	0.18	Glutathione peroxidase
**CNAG_02854**	-0.84	1.02	peroxiredoxin Q/BCP
**CNAG_03482**	3.13	-2.01	Thiol peroxidase
**CNAG_04388**	4.24	-3.14	mitochondrial manganese superoxide dismutase
**CNAG_04981**	2.82	-4.40	Catalase 1
**CNAG_05015**	3.31	-1.90	catalase 4
**CNAG_05256**	0.48	-1.76	catalase 2
**CNAG_05847**	4.78	-2.27	Thioredoxin reductase
**CNAG_06287**	0.12	0.06	Glutathione peroxidase
**CNAG_06917**	-1.70	1.68	thiol-specific antioxidant protein 3
**CNAG_07032**	2.31	-1.86	peroxiredoxin 5, atypical 2-Cys peroxiredoxin
**CNAG_07724**	0.68	-0.48	Metal-binding regulatory protein CUF1

Table S1: Expression of antioxidants in the WT oxidative stress response and differential expression in *hog1*Δ.

Transcripts listed under the GO term "antioxidant activity" are shown. Log2(fold-change) in expression was calculated using DEseq2 for the indicated pairwise comparison of samples.

bioRxiv preprint doi: https://doi.org/10.1101/2025.05.19.654802; this version posted May 23, 2025. The copyright holder for this preprint (which was not certified by peer review) is the author/funder, who has granted bioRxiv a license to display the preprint in perpetuity. It is made available under aCC-BY 4.0 International license.

Gene ID	log2(fold-change) in Expression		Gene Description
	WT (ROS-treated vs. untreated)	*gcn2*Δ vs. WT (during ROS)	
**CNAG_00058**	-0.63	2.12	T-complex protein 1 subunit epsilon
**CNAG_00060**	0.91	1.07	chaperone regulator
**CNAG_00447**	-0.71	2.31	T-complex protein 1 subunit beta
**CNAG_00510**	-1.53	1.78	prefoldin subunit 2
**CNAG_00619**	-0.31	-0.77	tubulin folding cofactor C
**CNAG_00987**	-1.06	1.33	prefoldin subunit
**CNAG_01110**	-1.01	1.02	co-chaperone
**CNAG_01343**	0.38	0.31	ATP-dependent Clp protease ATP-binding subunit ClpX
**CNAG_01568**	-0.21	2.14	T-complex protein 1 subunit alpha
**CNAG_01636**	-0.38	-0.81	peptidyl-prolyl cis-trans isomerase-like 1
**CNAG_01696**	1.13	3.17	chaperone DnaJ, variant
**CNAG_01881**	-0.46	1.92	molecular chaperone GrpE
**CNAG_01899**	-0.60	1.10	prefoldin alpha subunit
**CNAG_02440**	-0.37	1.90	cation-transporting ATPase
**CNAG_02492**	-0.66	-0.82	peptidyl-prolyl cis-trans isomerase-like 3
**CNAG_02500**	0.39	2.13	calnexin
**CNAG_02574**	-1.35	-1.02	cofactor D
**CNAG_02710**	-0.64	2.15	T-complex protein 1 subunit gamma
**CNAG_02736**	-0.79	2.01	T-complex protein 1 subunit theta
**CNAG_03459**	-0.50	2.54	T-complex protein 1 subunit delta
**CNAG_03486**	0.52	-0.36	peptidyl-prolyl cis-trans isomerase B
**CNAG_03621**	-0.57	2.06	Cyclophilin A
**CNAG_03627**	0.89	0.65	Cyclophilin A
**CNAG_03681**	-0.27	-0.21	peptidylprolyl isomerase domain and WD-repeat protein 1
**CNAG_03891**	-0.02	2.41	hsp60-like protein
**CNAG_03892**	0.01	2.63	chaperonin GroES
**CNAG_03944**	-0.13	2.92	chaperone regulator
**CNAG_04171**	1.04	-1.06	hypothetical protein
**CNAG_04304**	-0.22	2.33	T-complex protein 1 subunit zeta
**CNAG_04823**	-1.63	0.29	peptidyl-prolyl cis-trans isomerase H
**CNAG_04986**	-0.76	1.11	prefoldin subunit 1
**CNAG_05107**	-1.23	1.29	prefoldin subunit 4
**CNAG_05199**	0.80	1.87	chaperone DnaK
**CNAG_05252**	-0.28	0.61	chaperone regulator
**CNAG_05483**	-0.17	0.06	prefoldin beta subunit

bioRxiv preprint doi: https://doi.org/10.1101/2025.05.19.654802; this version posted May 23, 2025. The copyright holder for this preprint (which was not certified by peer review) is the author/funder, who has granted bioRxiv a license to display the preprint in perpetuity. It is made available under aCC-BY 4.0 International license.

**CNAG_06106**	2.84	2.00	chaperone regulator
**CNAG_06150**	0.69	2.41	hsp90-like protein
**CNAG_06927**	0.04	1.97	peptidyl-prolyl isomerase G (cyclophilin G)
**CNAG_07346**	-0.11	2.15	T-complex protein 1 subunit eta

Table S2: Expression of protein folding genes in the WT oxidative stress response and differential expression in *gcn2*Δ.

Transcripts listed under the GO term "protein folding" are shown. Log2(fold-change) in expression was calculated using DESeq2 for the indicated pairwise comparison of samples.

bioRxiv preprint doi: https://doi.org/10.1101/2025.05.19.654802; this version posted May 23, 2025. The copyright holder for this preprint (which was not certified by peer review) is the author/funder, who has granted bioRxiv a license to display the preprint in perpetuity. It is made available under aCC-BY 4.0 International license.

Gene ID	Log2(fold-change) in Expression		Gene Description
	WT (ROS-treated vs. untreated)	*hog1*Δ (ROS-treated vs. untreated)	
**CNAG_01005**	1.30	-0.25	glutaredoxin
**CNAG_01220**	1.18	0.24	mitochondrial FAD-linked sulfhydryl oxidase ERV1
**CNAG_02399**	0.86	-0.54	glutathione-disulfide reductase
**CNAG_02433**	0.82	0.04	thiol oxidase
**CNAG_02496**	1.21	0.64	hypothetical protein
**CNAG_03848**	1.28	0.08	glutathione transferase

Table S3: Expression of protein disulfide reductases that failed to be upregulated in *hog1*Δ ROS response.

Transcripts that were upregulated in the WT ROS response, but not the *hog1*Δ ROS response, and fall under the GO term "protein-disulfide reductase" are shown. Log2(fold-change) in expression was calculated using DEseq2 for the response of the indicated strains.

bioRxiv preprint doi: https://doi.org/10.1101/2025.05.19.654802; this version posted May 23, 2025. The copyright holder for this preprint (which was not certified by peer review) is the author/funder, who has granted bioRxiv a license to display the preprint in perpetuity. It is made available under aCC-BY 4.0 International license.

Gene ID	Log2(fold-change) in Expression			Gene Description
	WT (ROS-treated vs. untreated)	*hog1*Δ vs. WT (during ROS)	*gcn2*Δ vs. WT (during ROS)	
**CNAG_00663**	2.69	-1.42	-0.94	hypothetical protein
**CNAG_00668**	1.77	-2.10	-0.93	hypothetical protein
**CNAG_01272**	2.75	-3.85	-3.10	hypothetical protein
**CNAG_01506**	1.24	-2.76	-2.60	hypothetical protein
**CNAG_01704**	1.17	-2.73	-1.97	serine/threonine protein kinase
**CNAG_02156**	2.72	-2.41	-0.94	hypothetical protein
**CNAG_02510**	2.51	-2.76	-0.97	hypothetical protein
**CNAG_02820**	4.33	-4.21	-2.61	serine/threonine protein kinase
**CNAG_03019**	0.47	-0.93	-0.91	long-chain acyl-CoA synthetase
**CNAG_03135**	0.18	-1.95	-2.45	hypothetical protein
**CNAG_03223**	-0.36	-1.28	-1.79	hypothetical protein
**CNAG_03782**	7.45	-4.98	-4.21	hypothetical protein
**CNAG_03783**	5.00	-4.34	-2.36	hypothetical protein
**CNAG_04016**	1.38	-3.83	-1.35	Identified spore protein 5
**CNAG_04197**	2.85	-2.07	-1.09	CMGC/DYRK/YAK protein kinase
**CNAG_04272**	1.09	-1.14	-1.62	CAMK/CAMK1 protein kinase
**CNAG_04478**	2.49	-2.32	-0.81	hypothetical protein
**CNAG_04514**	0.85	-1.81	-1.32	CMGC/MAPK protein kinase
**CNAG_04606**	4.82	-3.05	-2.05	hypothetical protein
**CNAG_04634**	1.91	-2.59	-2.05	hypothetical protein
**CNAG_04681**	1.20	-2.30	-1.80	transmembrane protein, putative
**CNAG_04755**	1.62	-1.78	-0.87	STE/STE11/BCK1 protein kinase
**CNAG_04861**	-0.25	-0.92	-1.39	transglycosylase SLT domain-containing protein
**CNAG_04874**	-0.15	-0.86	-1.17	hypothetical protein
**CNAG_05439**	1.48	-1.64	-0.88	CAMK/CAMK1/CAMK1-CMK protein kinase
**CNAG_05654**	5.71	-4.62	-1.71	hypothetical protein
**CNAG_05818**	1.53	-2.12	-1.62	Chitin synthase
**CNAG_05836**	0.93	-2.73	-0.98	alpha 1,6-mannosyltransferase
**CNAG_05847**	4.78	-2.27	-1.28	Thioredoxin reductase
**CNAG_05891**	4.23	-2.46	-1.12	TDG/mug DNA glycosylase
**CNAG_06161**	1.34	-1.48	-0.83	hypothetical protein
**CNAG_06499**	3.69	-2.90	-1.35	hypothetical protein
**CNAG_06658**	4.49	-4.26	-2.13	rhomboid family membrane protein

bioRxiv preprint doi: https://doi.org/10.1101/2025.05.19.654802; this version posted May 23, 2025. The copyright holder for this preprint (which was not certified by peer review) is the author/funder, who has granted bioRxiv a license to display the preprint in perpetuity. It is made available under aCC-BY 4.0 International license.

**CNAG_06835**	2.43	-1.30	-1.20	glucosidase

Table S4: Stress response factors with lower expression in *hog1*Δ and *gcn2*Δ, relative to WT, during oxidative stress.

Transcripts with decreased expression in *hog1*Δ and *gcn2*Δ fall under the GO term "cellular response to stress" are shown. Log2(fold-change) in expression was calculated using DEseq2 for the indicated pairwise comparisons of samples.

bioRxiv preprint doi: https://doi.org/10.1101/2025.05.19.654802; this version posted May 23, 2025. The copyright holder for this preprint (which was not certified by peer review) is the author/funder, who has granted bioRxiv a license to display the preprint in perpetuity. It is made available under aCC-BY 4.0 International license.

Gene ID	Log2(fold-change) in Expression			Gene Description
	WT (ROS-treated vs. untreated)	*hog1*Δ vs. WT (during ROS)	*gcn2*Δ vs. WT (during ROS)	
**CNAG_00121**	2.02	-1.79	0.90	glycerol-3-phosphate dehydrogenase (NAD())
**CNAG_00154**	2.83	-2.47	1.11	oxidoreductase
**CNAG_01584**	4.00	-4.49	1.76	hydrolase
**CNAG_01604**	3.41	-1.33	2.28	TIGR01458 family HAD hydrolase
**CNAG_01605**	3.57	-3.26	1.18	rossman fold oxidoreductase
**CNAG_02519**	2.70	-0.97	1.53	mitochondria fission 1 protein
**CNAG_05573**	1.50	-1.17	0.86	cytochrome c oxidase assembly protein subunit 17
**CNAG_06868**	5.04	-4.22	1.39	Phosphopyruvate hydratase
**CNAG_06880**	1.99	-1.40	1.19	mitochondrial protein

Table S5: Selected targets with decreased expression in *hog1*Δ and increased expression in *gcn2*Δ, relative to WT during oxidative stress.

Selected transcripts from the list of 226 transcripts with decreased expression in *hog1*Δ and increased expression in *gcn2*Δ, relative to WT during oxidative stress. We selected transcripts with putative function in redox processes and detoxification. Log2(fold-change) was calculated using DEseq2 for the indicated pairwise comparisons of samples.

bioRxiv preprint doi: https://doi.org/10.1101/2025.05.19.654802; this version posted May 23, 2025. The copyright holder for this preprint (which was not certified by peer review) is the author/funder, who has granted bioRxiv a license to display the preprint in perpetuity. It is made available under aCC-BY 4.0 International license.

Gene ID	Log2(fold-change) in Expression					Gene Description
	WT (ROS-treated vs. untreated)	(ROS-treated mutant vs. ROS-treated WT)				
		hog1Δ	*gcn2Δ*	*hog1Δgcn2Δ* #9	*hog1Δgcn2Δ* #11	
**CNAG_00098**	2.13	-0.55	-0.47	-1.39	-1.31	palmitoyl-protein thioesterase
**CNAG_00318**	0.32	-0.49	0.12	-0.98	-0.87	arp2/3 complex 16 kda subunit
**CNAG_00450**	2.28	0.71	-0.76	-1.64	-1.47	3-isopropylmalate dehydrogenase
**CNAG_00749**	3.03	0.08	-1.38	-4.60	-4.95	alternative sulfate transporter
**CNAG_00905**	1.10	-0.69	0.08	-0.90	-1.31	MFS transporter
**CNAG_01489**	0.28	-0.54	-0.16	-1.41	-1.28	maltose O-acetyltransferase
**CNAG_03245**	1.69	-0.75	-0.03	-2.03	-1.94	glucose-6-phosphate dehydrogenase
**CNAG_03666**	0.87	-0.35	-0.64	-0.88	-1.22	acyl-CoA dehydrogenase
**CNAG_03920**	1.98	0.41	-0.60	-1.51	-1.43	isocitrate dehydrogenase, NADP-dependent
**CNAG_04013**	-0.40	-0.70	0.55	-0.97	-1.10	potassium channel protein
**CNAG_04765**	1.64	-0.59	0.23	-1.11	-0.94	ARP2/3 complex 21 kDa subunit
**CNAG_04898**	0.97	-0.35	-0.15	-0.95	-0.94	MFS transporter
**CNAG_05737**	2.24	-0.63	-0.53	-1.86	-1.77	oligopeptide transporter 8
**CNAG_06890**	0.91	-0.18	-0.26	-1.33	-1.79	membrane transporter
**CNAG_07582**	1.11	-0.10	0.18	-1.13	-1.27	dynein light chain roadblock-type

Table S6: Selected targets with uniquely lower expression in *hog1Δgcn2Δ*, with respect to WT during oxidative stress.

Selected transcripts from the list of 314 transcripts with decreased expression in *hog1Δgcn2Δ*, with respect to WT during oxidative stress, that did not reach thresholds for differential expression in single knockouts. We selected transcripts involved in metabolic processes, ion transport, and regulation of the cytoskeleton, which appeared to be most prevalent in this gene list. The log2(fold-change) for the indicated pairwise comparisons is shown.

bioRxiv preprint doi: https://doi.org/10.1101/2025.05.19.654802; this version posted May 23, 2025. The copyright holder for this preprint (which was not certified by peer review) is the author/funder, who has granted bioRxiv a license to display the preprint in perpetuity. It is made available under aCC-BY 4.0 International license.

Gene ID	Log2(fold-change) expression in *hog1Δ* vs. WT (during oxidative stress)	Gene Description
**CNAG_00580**	0.90	translation initiation factor IF-2
**CNAG_01428**	1.49	Translation initiation factor 5A
**CNAG_01660**	0.92	translation initiation factor eif-2b subunit alpha
**CNAG_01709**	-1.54	translation initiation factor 5
**CNAG_02128**	1.19	translation initiation factor 3 subunit J
**CNAG_02657**	1.04	translation initiation factor 3 subunit G
**CNAG_04022**	-0.86	translation initiation factor
**CNAG_04260**	1.45	translation initiation factor eIF-2B subunit gamma
**CNAG_05007**	1.11	translation initiation factor eIF-2B subunit beta
**CNAG_05843**	-1.25	translation initiation factor 4E
**CNAG_07628**	0.96	translation initiation factor 3 subunit C
**CNAG_07778**	0.92	translation initiation factor 2 subunit 1

Table S7: Translation initiation factors with dysregulated expression in *hog1*Δ, relative to WT during ROS.

Among transcripts upregulated in *hog1*Δ vs. WT during oxidative stress, transcripts with names containing "translation initiation factor" were selected. Log2(fold-change) in expression was calculated using DESeq2.
